# Integrated genomic and DNA methylome analyses reveal epigenetic regulation of stevia glycoside biosynthesis in *Stevia rebaudiana*

**DOI:** 10.1093/hr/uhaf226

**Published:** 2025-09-02

**Authors:** Genxiang Bai, Chengcai Xia, Zihao Wang, Zhiqiang Xia, Ming Luo

**Affiliations:** State Key Laboratory of Plant Diversity and Specialty Crops, South China Botanical Garden, Chinese Academy of Sciences, Guangzhou 510650, China; School of Breeding and Multiplication (Sanya Institute of Breeding and Multiplication), Hainan University, Sanya, Hainan 572025, China; School of Tropical Agriculture and Forestry, Hainan University, Sanya, Hainan 572025, China; School of Breeding and Multiplication (Sanya Institute of Breeding and Multiplication), Hainan University, Sanya, Hainan 572025, China; School of Tropical Agriculture and Forestry, Hainan University, Sanya, Hainan 572025, China; School of Breeding and Multiplication (Sanya Institute of Breeding and Multiplication), Hainan University, Sanya, Hainan 572025, China; School of Tropical Agriculture and Forestry, Hainan University, Sanya, Hainan 572025, China; State Key Laboratory of Plant Diversity and Specialty Crops, South China Botanical Garden, Chinese Academy of Sciences, Guangzhou 510650, China; University of Chinese Academy of Sciences, Chinese Academy of Sciences, Beijing 100049, China

## Abstract

*Stevia rebaudiana,* a member of the Asteraceae family, is a sugar-yielding plant abundant in stevia glycosides (SGs), which are extensively applied in sweeteners and pharmaceuticals. Although DNA methylation has been implicated in the regulation of specialized metabolite synthesis, its specific involvement in SG metabolism remains insufficiently elucidated. In this study, a chromosome-level genome assembly of *S. rebaudiana* was generated, including 1436 Mb across 11 chromosomes. DNA methylation profiling indicated that the expression of UDP-glycosyltransferases may be regulated by CG methylation within gene bodies across distinct tissues of *S. rebaudiana*. In conclusion, this study not only delivers a high-quality reference genome for *S. rebaudiana* but also provides novel perspectives on the potential regulatory mechanisms by which DNA methylation influences SG biosynthesis.

## Introduction

Obesity and diabetes represent significant public health challenges, closely linked to high-sugar dietary patterns that adversely affect human health. Excessive intake of sugar not only contributes to calorie overload but also elevate insulin resistance, potentially culminating in the onset of type 2 diabetes [1]. In response to these concerns, the utilization of sweeteners characterized by low caloric and high sweetness has emerged as a pivotal strategy. *Stevia rebaudiana*, a botanical species indigenous to Paraguay and a member of the Asteraceae family, contains leaves enriched with stevia glycosides (SGs). These SGs possess an intense sweetness, estimated at 150–300 times that of sucrose, while delivering merely one-three hundredth of the caloric content [2]. As a result, SGs have been recognized as a highly promising natural sweetening alternative. In recent years, the cultivation of stevia has expanded across Asia, North America, and Europe, reflecting increasing public attention to health-conscious dietary practices, Consequently, it has become a common additive in both food products and dietary supplements [3].

Epigenetic regulation is defined as heritable modifications in chromatin architecture and molecular characteristics that occur independently of changes in the DNA sequence [4, 5]. This regulatory framework encompasses diverse mechanisms, including DNA methylation, histone modifications, chromatin remodeling, and the activity of noncoding RNA [6]. Such processes are indispensable for plant development, growth responses to biotic and abiotic stressors, as well as the production of secondary metabolites [7–10]. Cytosine methylation (mC), one of the most widespread and evolutionarily conserved epigenetic alterations in eukaryotes, serves critical functions in preserving genome integrity, modulating gene expression regulation, and silencing transposable elements (TEs) [11]. Moreover, mC has been identified as a key determinant in the biosynthetic pathways of plant-derived secondary metabolites [12]. The maintenance of CG methylation is mediated by METHYLTRANSFERASE 1 (MET1), while CHG methylation is controlled through the action of CHROMOMETHYLASE 3 (CMT3) and METHYLTRANSFERASE 2 (CMT2). Asymmetric CHH methylation is preserved primarily through the activity of DOMAIN-REARRANGED METHYLTRANSFERASE 2 (DRM2) and METHYLTRANSFERASE 2 (CMT2) [13, 14]. Additionally, active DNA demethylation is orchestrated by specific enzymes such as REPRESSOR OF SILENCING 1 (ROS1), DEMETER (DME), and DEMETER-LIKE (DML) proteins, which collectively modulate the global DNA methylation landscape [15, 16].

A plant’s specialized metabolites, while not essential for fundamental physiological processes, are indispensable for survival, reproduction, defense, and interactions with surrounding organisms [17, 18]. Recent studies have demonstrated that genome-wide alterations in DNA methylation patterns exert a significant influence on the biosynthesis and accumulation of these metabolites [19–21]. In carnation, elevated methylation within gene bodies or promoter has been associated with the downregulation of genes involved in the synthesis of anthocyanins and carotenoids [22]. Moreover, DNA methylation modulates gene expression across distinct plant tissues, and diminished genome-wide methylation in *Salvia miltiorrhiza* correlates with an increase in tanshinone content [12]. In tea roots, methylation within the CS gene body has been shown to enhance catechin accumulation [23]. Application of DNA methylation inhibitor 5-azacytidine (5′-Aza) in medicinal plants results in reduced global methylation, activation of genes associated with the biosynthesis of specialized metabolites, and subsequent enhancement of their accumulation [10]. These findings highlight the regulatory significance of DNA methylation in regulating plant secondary metabolite production. Nonetheless, the precise mechanisms by which DNA methylation governs SG biosynthesis remain insufficiently understood.

SGs are tetracyclic diterpenoid compounds derived from *S. rebaudiana* leaves, and their biosynthetic pathway exhibits a high degree of similarity to that of gibberellins (GAs). Initially, isopentenyl pyrophosphate (IPP) and dimethylallyl diphosphate (DMAPP) are synthesized via the cytosolic mevalonate pathway [24, 25]. These intermediates are subsequently converted into *ent*-copalyl diphosphate through the catalytic action of *ent*-copalyl diphosphate synthase (*ent*-CPS). The resulting product is then transformed into *ent*-kaurene by *ent*-kaurene synthase (*ent*-KS), followed by is oxidation to *ent*-kaurenoic acid by *ent*-kaurene oxidase (*ent*-KO) [26, 27]. In plants, GAs are synthesized from *ent*-kaurene through the enzymatic function of *ent*-kaurene oxidase (*ent*-KAO). In contrast, in *S. rebaudiana*, *ent*-kaurenoic acid undergoes hydroxylation at the C13 position by *ent*-kaurenoic acid hydroxylase (*ent*-KAH), resulting in the formation of steviol. Subsequent steps involve the glycosylation of steviol into variety of SGs by distinct UDP-glycosyltransferases (UGTs) [28]. For instance, SrUGT85C2 catalyzes the conversion of steviol to steviolmonoside, whereas UGT74G1 facilitates the transformation of steviolbioside into stevioside (Stev) [29, 30]. The glycosyltransferase UGT76G1 in *S. rebaudiana* has been structurally elucidated, including its protein architecture and active site configuration, and is responsible for the conversion of Stev into rebaudioside A (Reb A) [31, 32]. Furthermore, OsUGT91C1 has been shown to catalyze the formation of β (1 → 2) glycosidic bond, enabling glycosylation at both the positions R1 and R2 [33]. Despite the function characterization of several UGTs, numerous UGT genes implicated in SG biosynthesis remain functionally uncharacterized.

To investigate the regulatory role of DNA methylation in SG biosynthesis in *S. rebaudiana*, targeted metabolite profiling, genomic analysis, nanopore-based whole-genome sequencing, and transcriptomic data were comprehensively integrated. A high-quality, chromosome-level genome assembly for *S. rebaudiana* was constructed using a combination of PacBio, Hi-C, and Illumina sequencing technologies. Targeted metabolic assays indicated that SGs predominantly accumulate in leaf tissues. Methylation profiling via nanopore sequencing revealed distinct DNA methylation landscapes across different tissue types. Through the integration of transcriptomic and DNA methylomes datasets, 118 differentially expressed genes (DEGs) associated with differentially methylated regions (DMRs) were identified, providing valuable insights into tissue-specific epigenetic regulation in SG biosynthesis. Notably, two SG biosynthesis-associated genes, *SrUGT85C2-8* and *SrUGT76G1-11*, were found to harbor hypomethylated CG sites within their gene bodies, a feature correlated with markedly elevated expression levels in stems and leaves. Moreover, treatment with 5′-Aza not only promoted SG accumulation but also repressed the expression of DNA methyltransferase genes, indicating that reduced DNA methylation may be a pivotal contributor to enhanced SG biosynthesis. These findings reveal a potential regulatory function of CG methylation within gene bodies in modulating SG biosynthesis and emphasize the importance of epigenetic modifications in the transcriptional regulation of related genes.

## Result

### The SG content varies across different tissues in *S. rebaudiana*

**Figure 1:**
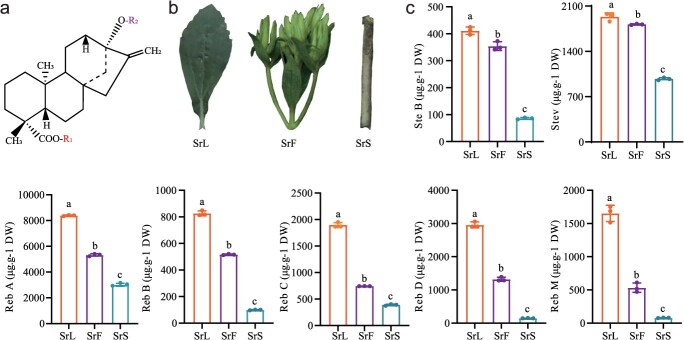
SG content in different tissues of *S. rebaudiana*. (a) The structural formula of SGs are presented. (b) The phenotypic characteristics of the leaves, flowers, and stems of *S. rebaudiana* are depicted. (c) The content of SG in different tissues during the early flowering stage is quantified: Ste B, Stev, Reb A, Reb B, Reb C, Reb D, and Reb M in *S. rebaudiana* leaves (SrL), S. rebaudiana flowers (SrF), *S. rebaudiana* stems (SrS). The data in (c) were subjected to one-way ANOVA followed by the least significant difference test. Bars labeled with different letters indicate significant differences at p ≤ 0.05. (The species *S. rebaudiana* and other series in italics.)

SGs are tetracyclic diterpene compounds featuring glucose, rhamnosyl, and xylosyl moieties attached at the C13 and C19 positions (Fig. 1a). To assess SG content in various tissues of *S. rebaudiana* during the early flowering stage, targeted metabolic analysis profiling was conducted using liquid chromatography–tandem mass spectrometry in multiple reaction monitoring (MRM) mode (Fig. 1b). Multiple SGs were identified across the examined tissues, including Stev, Stev B, Reb A, Reb B, Reb C, Reb D, and Reb M. Quantitative analysis revealed that SG levels were markedly elevated in leaves compared to flowers and stems (Fig. 1c), with significant differences also detected between flowers and stems (Fig. 1c). These findings indicate a tissue-specific pattern of SG distribution, with leaves exhibiting the highest concentration.

### Genome assembly and annotation of *S. rebaudiana*

**Figure 2:**
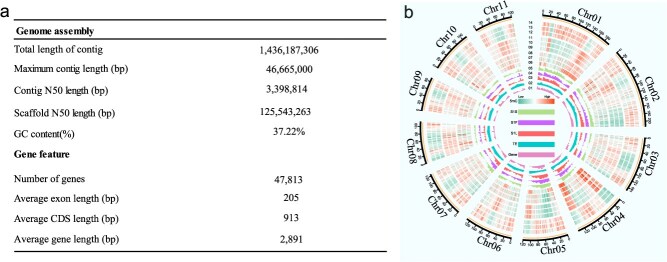
Genomic features of *S. rebaudiana* assembly and annotation. (a) Key information about the *S. rebaudiana* genome is presented in the table. (b) The following genomic features are visualized through the circos plot: 1. Gene density; 2. Transposable element (TE) density; 3-14. DNA methylation levels of CG, CHG, and CHH contexts across three samples (SrF, SrL, and SrS).

To investigate the genetic foundation of SG biosynthesis in *S. rebaudiana*, the genome of the ‘Zhongke No. 1’ variety was sequenced and assembled using Oxford Nanopore technologies, complemented with Bionano optical mapping and Hi-C sequencing. The resulting genome assembly spanned 1436 Mb, with a contig N50 of 3.39 Mb (Fig. 2a). Furthermore, through the application of Hi-C double-terminal read clustering, sequencing, and orientation, a total of 1436 Mb of scaffolds were accurately anchored onto 11 pseudo-chromosomes (2*n* = 22), encompassing ~99.99% of the assembled sequences (Fig. 2a and b, Fig. S1a). Assessment using the Benchmarking Universal Single-Copy Orthologs (BUSCO) framework indicated that 96.03% (1550 of 1614) of the expected single-copy orthologs were successfully identified in the genome assembly (Fig. S2b, Table S1).

Homology-based, *de novo*, and transcriptome-assisted strategies were integrated to predict gene models, resulting in the identification of 47 813 protein-coding genes with an average gene length of 2891 bp and a mean coding sequence length of ~913 bp (Fig. 2a). Among the annotated genes, 39 980 (83.62%) of the genes were successfully mapped to entries in the Kyoto Encyclopedia of Genes and Genomes (KEGG), Clusters of Orthologous Groups, and Gene Ontology (GO) databases (Table S2). This elevated annotation rate indicates that the genome retains a broadly conserved gene function repertoire. Collectively, these findings reflect the high completeness and precision of the *S. rebaudiana* genome, thereby establishing a robust basis for future genetic and functional investigations.

### Evolutionary history and whole-genome duplication of *S. rebaudiana*

To investigate the evolutionary trajectory of *S. rebaudiana*, a phylogenetic analysis was conducted using 15 additional sequenced genomes, these included *Amborella trichopoda*, *Elaeis guineensis*, *Ananas comosus*, *Oryza sativa*, *Pennisetum alopecuroides*, *Sorghum bicolor*, *Arabidopsis thaliana*, *Zea mays*, *Passiflora edulis*, *Vitis vinifera*, *Camellia sinensis*, *Cichorium endivia*, *Lactuca sativa*, *Helianthus annuus*, and *Smallanthus sonchifolius.* Phylogenetic relationships were inferred based on 963 single-copy orthologous genes, confirming the evolutionary associations among these species. Five Asteraceae species (*S. rebaudiana*, *S. sonchifolius*, and *H. annuus, C. endivia*, and *L. sativa*) clustered into a monophyletic clade, which diverged ~34 million years ago (Mya). Within this clade, *S. rebaudiana*, *H. annuus*, *and S. sonchifolius* were found to comprise a distinct subgroup, Molecular clock analysis estimated that *S. rebaudiana* diverged from *H. annuus* and *S. sonchifolius* ~21 Mya. A total of 47 813 *S. rebaudiana* genes were assigned to 21 485 gene families based on sequence homology. Comparative genomic analysis revealed that 11 174 gene families were conserved across other plant species, while 2849 gene families appeared to be unique to *S. rebaudiana* (Fig. S2a). These lineage-specific genes families were predominantly enriched in biosynthetic pathways, such as ‘Metabolism of terpenoids and polyketides’, ‘Sesquiterpenoid and triterpenoid biosynthesis’, ‘Glycosyltransferases’, ‘Terpenoid backbone biosynthesis’, ‘Monoterpenoid biosynthesis,’, and ‘Biosynthesis of other secondary metabolites’ (Fig. S2b, Table S3). Additionally, 3783 gene families were identified as expanded and 2127 as contracted.

**Figure 3:**
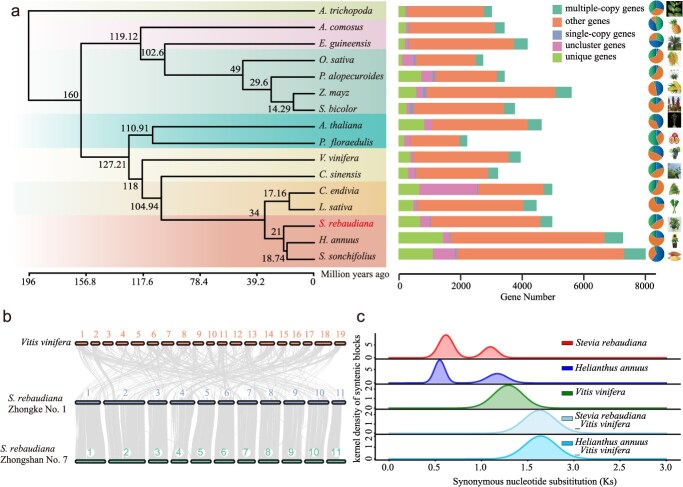
Phylogenetic tree and comparative genomic analysis of *S. rebaudiana*. (a) Phylogenetic relationships were constructed using single-copy genes shared among 16 species. (b) Genome collin-earity analysis was performed between *S. rebaudiana* (Zhongke No. 1), *S. rebaudiana* (Zhongshan No. 7), and *Vitis vinifera*. (c) The distribution of Ks values for segmental duplication gene pairs in *S. rebaudiana* provides evidence for a WGD event in the Lamiales order and an ancient WGT in core eudicots.

To elucidate the polyploidization history of *S. rebaudiana*, the well-characterized polyploidy events in grape were employed as a reference, and homology analysis was performed in comparison with sunflower. Concurrently, orthologous and paralogous genomic regions were identified in these species. In grape, six genomic regions were found to correspond to homologous segments in both *S. rebaudiana* and sunflower (Fig. S3a and b), suggesting that additional duplication events occurred in both species following the core eudicot whole-genome triplication (WGT). Comparison analysis between the genomes of sunflower and *S. rebaudiana* revealed relationships between 1:1 orthologous and paralogous genomic regions (Fig. S3c and d), implying both species share a more recent whole-genome duplication (WGD) event. Furthermore, one genomic region in sunflower was shown to align with a single orthologous region in *S. rebaudiana* (Fig. S3e). To further investigate these evolutionary events, distribution curves of synonymous nucleotide substitution rates (KS) were generated for collinear genomic regions in both *S. rebaudiana* and sunflower (Fig. 3b). In the sunflower genome, two distinct KS distribution peaks were observed: a younger peak at 0.55 corresponding to the most recent WGD event, and an older peak at 1.17, associated with the shared WGT event within the Asteraceae lineage. These results suggest that one genomic region in grape corresponds to six syntenic regions in *S. rebaudiana*, thereby supporting the occurrence of multiple duplication events. The *S. rebaudiana* genome similarly exhibited two peaks (0.61 and 1.10), corresponding to a WGD and a WGT event, respectively (Fig. 3c). Collectively, the findings indicate that both sunflower and *S. rebaudiana* underwent an ancient triplication event (WGT-γ) common to several species, a WGT event specific to the Asteraceae family, and a more recent WGD event unique to these two species*.*

### mCG likely influences methylation changes in *S. rebaudiana*

DNA methylation sequencing was performed using the ONT PromethION platform on leaves (SrL), flowers (SrF), and stem (SrS) tissues of *S. rebaudiana* to investigate methylation dynamics across distinct organs. The average data yield was 32.97 Gb for leaves, 24.95 Gb for flowers, and 22.89 Gb for stems, with sequencing depth exceeding 15× for each tissue type (Fig. 2b, Table S4), thereby ensuring high data for subsequent analyses.

**Figure 4:**
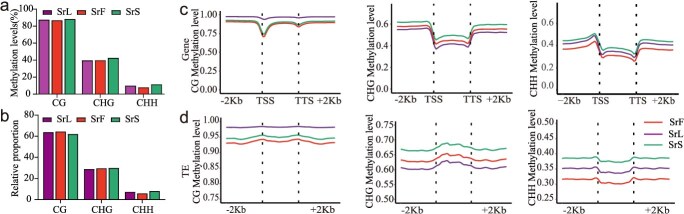
DNA methylation variations in different tissues of *S. rebaudiana*. (a) The average genome-wide DNA methylation levels in flower, leaf, and stem tissues of *S. rebaudiana* are categorized by CG, CHG, and CHH sequence contexts. (b) The relative proportions of methylated cytosines within CG, CHG, and CHH contexts are depicted for *S. rebaudiana*. (c, d) Metaplots illustrate the patterns and levels of DNA methylation across protein-coding genes and transposons in the three tissue types. These metaplots include annotations for transcription start sites (TSS) and termination sites (TTS).

The mean methylation levels for each tissue across different sequence contexts (CG, CHG, and CHH) were calculated genome-wide. The results demonstrated that stems exhibited higher methylation levels than flowers and leaves in all three sequence contexts (Fig. 4a). The relative proportions of methylation mirrored their overall methylation patterns for CHG and CHH, although a divergent trend was observed in CG methylation (Fig. 4b). Further analysis of CG methylation in genes and TEs revealed distinct distribution patterns compared to CHG and CHH methylation, with comparable levels detected in gene bodies, TEs, and their flanking regions. Leaf CG methylation was notably higher than that in stems and flowers. In contrast, CHG and CHH methylation levels were elevated in upstream and downstream regions relative to gene bodies and TEs, except for CHG methylation within TEs, where a progressive increase in methylation was observed from leaves to flowers to stems (Fig. 4c and d). DNA methylation in TE regions was specifically examined due to its known role in regulating TE activity. Compared to their adjacent flanking regions, TEs displayed increased CG and CHG methylation. Additionally, CHH methylation was found to be enriched near transcription start and termination sites relative to central TE regions, suggesting that a sophisticated regulatory mechanism may contribute to tissue-specific methylation patterns (Fig. 4d). These findings suggest that variation in methylation levels in *S. rebaudiana* may be influenced, at least in part, by differential mCG methylation.

**Figure 5:**
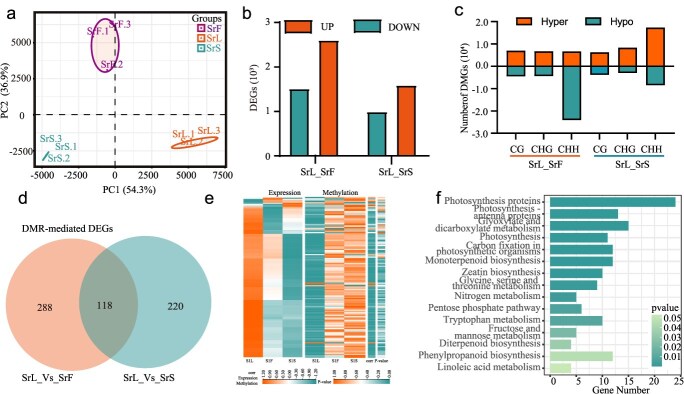
Association between CG context DMRs and DEGs. (a) Principal component analysis (PCA) demonstrates the consistency among the three biological replicates of SrL, SrF, and SrS. (b) The number of DEGs identified in the comparisons between SrL_vs_SrF and SrL_vs_SrS is presented in the following analysis. (c) The total number of SrL_vs_SrF and SrL_vs_SrS hyper- and hypo-DMGs in the CG, CHG and CHH contexts is shown in a bar plot. (d) The overlap between intersection of DMR-mediated DEGs with DMRs in the SrL_vs_SrF and SrL_vs_SrS groups, respectively, is shown in a Venn diagram. (e) The relationship between methylome and transcriptome is revealed through an integrated analysis. (f) The results of KEGG pathway enrichment analysis for all DMR-mediated DEGs are presented in a bar plot.

### Hyper-CG DNA methylation may affect gene expression

To examine the relationship between DNA methylation and gene expression across multiple tissues, transcriptome sequencing was performed on stem, leaf, and flower samples using the same materials employed for DNA methylome sequencing. Principal component analysis (PCA) revealed high consistency among replicates within each tissue type (Fig. 5a). DEG analysis identified 6635 DEGs in the SrL_SrF comparison (3124 upregulated and 3511 downregulated genes) and 4565 DEGs in SrL_SrS (1946 upregulated and 2719 downregulated genes) (Fig. 5b). Genes exhibiting differential DNA methylation within 2 kb upstream or downstream of the transcription start site were defined as DMR-associated genes (DMGs). DMGs were detected in all three tissues. Among the three methylation contexts, the CHH context yielded the highest number of DMGs, whereas the CG context showed the lowest. In the SrL_SrF and SrL_SrS comparisons, 4551 and 3832 hypermethylated genes were identified, respectively, in the CG context, while 6998 and 6257 hypomethylated genes were observed in the same comparisons. Similarly, in the CHG context, 4409 hypermethylated and 6738 hypomethylated genes were identified in SrL_SrF, and 3023 hypermethylated and 8349 hypomethylated genes in SrL_SrS. In the CHH context, 24 095 hypermethylated and 8509 hypomethylated genes were found in SrL_SrF, whereas 6636 hypermethylated and 17 367 hypomethylated genes were observed in SrL_SrS (Fig. 5c).

**Figure 6:**
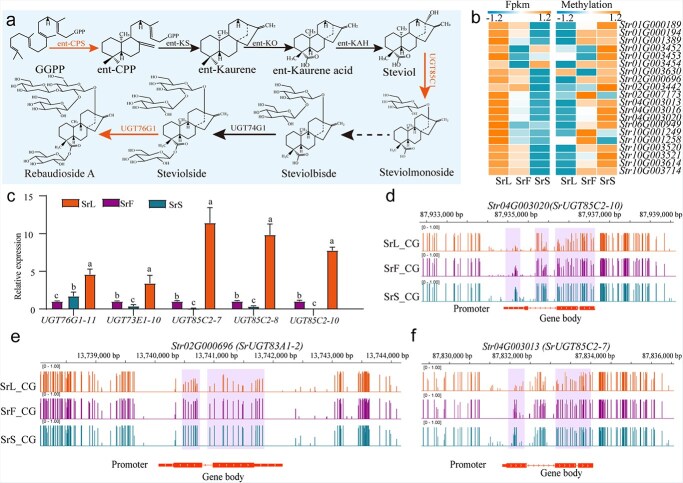
Decreased CG methylation contributes to SGs biosynthesis in *S. rebaudiana*. (a) The SGs biosynthesis pathway is depicted in a diagram. (b) Gene expression and DNA methylation levels of genes involved in the SGs biosynthesis pathway are presented. (c) The relative expression of genes associated with methylation in SGs biosynthesis is shown. (d-f) The genome browser displays the DNA methylation and expression levels of representative SGs biosynthesis - related genes are displayed in the genome browser, with methylation levels indicated by the height of the vertical bar on each track.

To elucidate the relationship between DNA methylation changes and gene expression, DMGs in the CG context and their associated DEGs were analyzed. A total of 406 DMGs were identified in SrL_SrF, and 338 in SrL_SrS, with 118 DMGs overlapping between the two groups (Fig. 5d, Table S5). Correlation analysis was subsequently conducted to assess the association between methylation levels and gene expression. The results revealed that >80% of DMR-mediated DEGs across tissues exhibited a negative correlation with their corresponding methylation levels (Fig. 5e). This inverse relationship was further visualized through heatmaps, suggesting a possible correlation between gene body methylation levels and gene expression. Additionally, functional enrichment analysis showed that all DMR-mediated DEGs were primarily involved in key biological pathways, including ‘Photosynthesis proteins’, ‘Photosynthesis - antenna proteins’, ‘Glyoxylate and dicarboxylate metabolism’, ‘Photosynthesis’, ‘Carbon fixation in photosynthetic organisms’, and ‘Monoterpenoid biosynthesis’ (Fig. 5f, Table S6). Among them, the monoterpenoid biosynthetic pathway includes that of SGs. Therefore, we speculate that the GC methylation process may be involved in the regulation of SGs synthesis.

### DNA methylation may be associated with UGT expression

To investigate the expression patterns of genes involved in the SG biosynthetic pathway across different tissues of *S. rebaudiana*, particular emphasis was placed on SrUGTs, enzymes known to contribute to SG structural variation (Fig. 6a). Transcriptome analysis identified a total of 296 (UGT) genes, among which 20 were DMR-mediated DEGs between the SrL_vs_SrF and SrL_vs_SrS comparisons (Table S7). Interestingly, these UGT genes exhibited a strong negative correlation (Cor ≤ −0.8) with DNA methylation levels (Fig. 6b), suggesting that reduced methylation is associated with increased gene expression in leaves. Notably, several *SrUGTs*, including *SrUGT76G1*, *SrUGT71E1*, *SrUGT72E1*, *SrUGT73C4*, *SrUGT74B1*, *SrUGT73E1*, *SrUGT83A1*, *SrUGT85A8*, *SrUGT85C1*, *SrUGT85C2*, and *SrUGT91D1*, showed substantially elevated expression in leaves compared to flowers and stems (Table S8). These results point to a potential regulatory role of CG methylation in modulating UGT gene expression.

**Figure 7:**
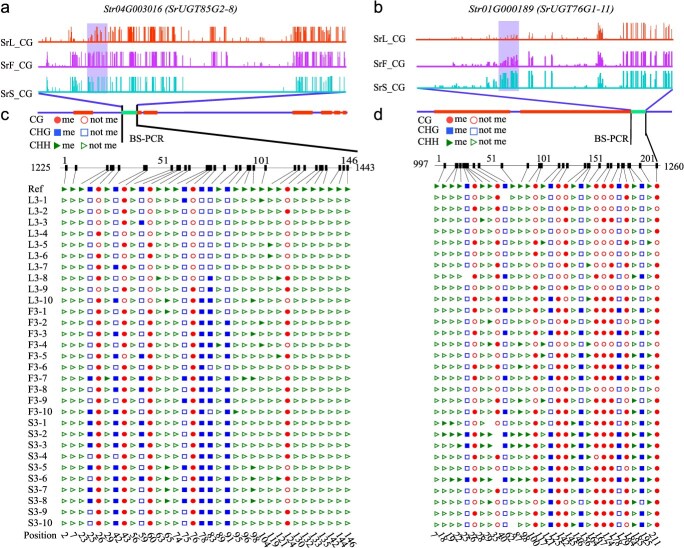
Illustrates the methylation patterns of *SrUGT* genes with DMG in SGs biosynthesis. (a, b) IGV display showing mCG methylation levels within the gene bodies of *SrUGT85C2-8* and *SrUGT76G1-11*. Regions with varying methylation levels are highlighted by purple boxes. (c) Bisulfite sequencing was employed to analyze the methylation status of the *SrUGT85C2-8* gene body. (d) Bisulfite sequencing analysis of the *SrUGT76G1-11* gene body, along with a schematic diagram of its structure. The coding sequences (exons) are represented as orange boxes, intronregions as blue boxes, and differentially methylated regions (DMRs) as green boxes. The tissues analyzed include leaf (L), stem (S), and flower (F).

Among the identified DMR-mediated DEGs, *SrUGT85C2* and *SrUGT76G1* were confirmed to be associated with the SG biosynthetic pathway (Fig. 6a). These enzymes exhibited reduced methylation across their gene regions and showed elevated expression levels specifically in leaves compared to flowers and stems. Methylation occurring within promoter and downstream regions has been recognized as a key factor in modulating gene activity. To exclude potential regulatory effects from these regions, methylation profiles were visualized using Integrative Genomics Viewer (IGV), which revealed no significant variations (Fig. 6b–f). The observed negative correlation between DNA methylation and gene expression in these UGTs is likely attributable to hypo-DMRs located within their gene bodies, a finding further corroborated by Nanopore sequencing data for *SrUGT85C2-8* and *SrUGT76G1-11* (Fig. 7a and b). To validate these observations, bisulfite polymerase chain reaction (BS-PCR) analysis was performed on *SrUGT85C2-8* and *SrUGT76G1-11*, which revealed reduced methylation in specific upstream leaf regions (−1225 to −1443 bp for *SrUGT85C2-8* and −977 to −1260 bp for *SrUGT76G1-11*) (Fig. 7). Additionally, both *SrUGT85C2-8* and *SrUGT76G1-11* exhibited substantially lower methylation levels in leaves than in stems and flowers (Fig. 7), supporting that CG-specific methylation may be associated with UGT gene expression in *S. rebaudiana*.

### Methylation inhibitor 5′-Aza promotes SG accumulation

To further investigate the association between SG content and DNA methylation, dynamic changes in SG levels were evaluated following treatment with 5′-Aza, a nucleoside methylation inhibitor. Application of 50 mM 5′-Aza resulted in a significant increase in the levels of SGs, including Ste B, Stev, Reb A, Reb B, and Reb C, in callus tissue, while concurrently reducing the expression levels of methyltransferase genes (Fig. S4a and c). Subsequent analysis revealed that the expression of several methyltransferase genes, namely *SrDRM3*, *SrDRM2-1*, *SrDRM2-2*, *SrCMT2-1*, *SrCMT2-2*, *SrDRM2-3*, *SrCMT2-4*, *SrMET1B*, *SrMET4*, and *SrCMT3-3*, was suppressed following treatment (Fig. S4b). Conversely, the expression levels of glycosyltransferase genes, such as *SrUGT76G1-19*, *SrUGT85C2-9*, *SrUGT85C2-8*, and *SrUGT73E1-15*, were elevated (Fig. S4c), suggesting a potential regulatory function of DNA methylation in SG biosynthesis.

## Discussion

A plant’s specialized metabolites are not directly involved in growth and development but are integral to stress responses [34]. The biosynthesis of these metabolites is generally activated under biotic and abiotic stress conditions [35]. Increasing research attention has elucidated the mechanisms underlying transcriptional regulation and epigenetic modification in plant secondary metabolism, as demonstrated in species such as *S. miltiorrhiza*, carnation, and pear [12, 36, 37]. In this study, the *S. rebaudiana* genome was assembled, and DNA methylomes, transcriptomes, and metabolite profiles were analyzed across different tissues to elucidate the regulatory function of DNA methylation in SG synthesis.

High-quality genome assemblies are essential for elucidating plant gene functions and advancing omics-based research [38]. In this study, a chromosome-level genome assembly of *S. rebaudiana* ‘Zhongke No. 1’ was generated using a hybrid sequencing strategy integrated with Hi-C scaffolding. The assembled genome spans 1436 Mb, with a contig N50 of 3.39 Mb (Fig. 2a). This assembly represents a substantial improvement over previous versions, which reported N50 values of 616.85 kb (1.16 Gb) and 841 kb (1.34 Gb) [27, 39]. Assessment using the BUSCO framework indicated 96.03% genomic completeness (Tables S1), establishing this assembly as a high-quality reference genome for *S. rebaudiana*. The resulting genome sequence serves as a valuable resource for studies of genetic diversity, functional genomics, and pan-genome evolution in *S. rebaudiana*. Additionally, it provides critical insights into evolutionary genomics and facilitates the identification of genes and metabolic pathways involved in SG biosynthesis.

Methylation modifications are predominantly localized within GC-rich and highly repetitive genomic regions, including centromeric repeats, ribosomal RNA-encoding loci, and TEs [7, 40]. In plants, DNA methylation occurs primarily in three sequence contexts, CG, CHG, and CHH [41], which are known to regulate growth and development and are modulated by both developmental and environmental factors [42]. Previous studies have demonstrated tissue-specific variation in methylation levels, which may be associated with the biosynthesis of specific metabolites. In celery, apigenin production is inversely associated with methylation levels in different tissues [19]. This relationship has also been found in tea plant species [23]. In our study, methylation levels increase progressively from flowers to leaves to stems during the early flowering stage, particularly in gene-coding and TE regions for CHG and CHH contexts (Fig. 4b and c). CG methylation is significantly higher than CHG and CHH and may contribute to tissue-specific methylation patterns (Fig. 4b and c). Furthermore, the overall methylation levels were observed to exceed those reported by conventional WGBS, such as with apple [43], orange [16] and pear [44], likely attributable to the characteristics of nanopore sequencing [45, 46].

Methylation within gene body flanking regions and TE regions is recognized as a key component of gene expression regulation [7]. In most plant species, flanking regions typically exhibit higher methylation levels than gene bodies, whereas TE regions generally display greater methylation than their adjacent flanking sequences [16, 43]. In this study, consistent methylation patterns were observed for CHH and CHG contexts in gene bodies and for CHG in TE regions (Fig. 4b and c), suggesting evolutionary conservation. However, CHH methylation in TE regions was found to resemble that in gene bodies, with higher levels detected in TEs than in their flanking regions (Fig. 4b and c). Additionally, no significant differences were detected in GC methylation levels between TE and gene regions or their corresponding flanking sequences (Fig. 4b and c), implying the presence of a more complex regulatory mechanism. A comparable pattern has been identified in CG methylation within gene bodies during the ripening process of pear and orange fruit [16, 44].

SGs constitute the primary bioactive components in *S. rebaudiana* leaves, with Stev and Reb A comprising ~65%–70% of the total SG content. Their accumulation substantially influences the commercial value of stevia, thereby highlighting the importance of understanding how gene methylation regulates SG biosynthesis [28]. Previous studies have demonstrated that CHH context DNA methylation plays a key role in the epigenetic regulation of gene activity [47]. Current studies indicate that CG-type methylation in gene body regions is not associated with transcriptional gene silencing [7]. However, some studies have shown that CG methylation in gene bodies plays a role in regulating the biosynthesis of plant secondary metabolites. For example, in tea and parsley, elevated CG methylation levels across tissues have been correlated with suppressed gene expression [19, 23], while in *S. miltiorrhiza*, increased gene body methylation has been linked to reduced tanshinone accumulation [12]. In this study, DMR-associated DEG analysis revealed that 21 UGT genes exhibited inverse correlations between expression and gene body methylation across tissues (Fig. 6b). Moreover, no notable alterations were detected in methylation patterns within upstream or downstream regions (Fig. 6). BS-PCR further confirmed hypomethylation in the gene body regions of the leaf-expressed glycoside synthesis-related genes *SrUGT76G1-11* and *SrUGT85C2-8* (Fig. 7), suggesting that their elevated expression may be associated with reduced CG methylation within these domains. Moreover, experimental results indicated that treatment with 5′-Aza not only enhanced UGT gene expression and SG accumulation but also suppressed the expression of methyltransferase genes (Fig. S4), thereby reinforcing the potential regulatory role of DNA methylation in SG biosynthesis.

## Materials and methods

### Plant materials and genome sequencing

The *S. rebaudiana* variety ‘Zhongke No. 1’ was selected for *de novo* genome sequencing and cultivated in a trial field located in Guangzhou, Guangdong, China (23°11′29.38″N, 113°21′45.97″E). Fresh and tender tissues were harvested at the initial flowering stage, rapidly frozen in liquid nitrogen, and stored at −80°C for subsequent extraction of DNA, RNA, and SGs. Genomic DNA isolation, quality assessment, and sequencing were conducted by Hainan Chuangsiji Bioinformatics Technology. Simultaneously, the same biological samples used for genome sequencing were also collected for methylation and transcriptomic analyses. These included SrL, SrF, and SrS at the initial flowering stage.

To investigate the effects of a DNA methylation inhibitor, a solid medium containing 50 mM 5′-Aza was prepared by supplementing basal MS medium with 30 g/l sucrose and 1.0 mg/l TDZ. As the control (CK), *S. rebaudiana* callus was cultured on an identical medium lacking 5′-Aza. The 5′-Aza-treated callus was maintained under continuous illumination at 25°C for 7 days before analysis.

### Genome assembly and evaluation

The raw data generated from leaf tissues through Nanopore sequencing were initially error-corrected and assembled *de novo* using NextDenovo v2.3.1 (https://github.com/Nextomics/NextDenovo) [45]. Duplicate sequences were subsequently eliminated using Purge_dups (https://github.com/dfguan/purge_dups). The resulting genome assembly was further refined by polishing with both Illumina short reads and Nanopore long reads via Nextpolish v1.3.1, employing default parameters to enhance sequence fidelity. Hi-C data were utilized to facilitate chromosome-level assembly by anchoring scaffolds to chromosomal positions. The completeness and structural integrity of the final assembly were evaluated using BUSCO (https://anaconda.org/bioconda/busco), which provided standard metrics for genome quality assessment.

TEs were identified through a combination of *de novo* and homology-based prediction approaches. The genome was initially premasked using RepeatModeler v1.0.9 [48] (http://www.repeatmasker.org/RepeatModeler) to construct a customized repeat element library. RepeatMasker v3.3.0 (http://www.repeatmasker.org/) was subsequently employed to detect repetitive sequences by referencing both the RepBase database and the generated repeat library. Annotation of protein-coding genes was performed using the MAKER pipeline, and gene functions were characterized through GO and KEGG pathway enrichment analyses.

Single-copy orthologous genes were identified using OrthoFinder, and a phylogenetic tree was constructed based on these genes. This tree was subsequently transformed into a time-calibrated phylogeny utilizing the R8S program. Divergence times among species were estimated through TimeTree [49] (http://www.timetree.org/). Expansion and contraction of gene families were assessed using the Computational Analysis of Gene Family Evolution [50] software package. OrthoVenn2 (https://orthovenn2.bioinfotoolkits.net/home) was employed to detect orthologous gene cluster clades specific to the Asteraceae family. Collinearity blocks between *S. rebaudiana* and *H. annuus*, were visualized via MCScanX [51] (JCVI), and WGD events were inferred using the NG86 algorithm.

### SG content determination

SG was extracted from various tissues utilizing 80% methanol (v/v), followed by filtration through a 0.22 μm membrane filter. Quantification and analysis of SGs were performed employing ACQUITY™ UHPLC operating in MRM mode with a dwell time of 0.025 s, and the resulting data were processed using Xcalibur software.

### Methylation analysis

Nanopore sequencing files were consolidated into a single FAST5 file through the utilization of the FAST5 API provided by Oxford Nanopore Technologies (https://github.com/nanoporetech/ont_fast5_api). Base calling of the FAST5 files was conducted using Guppy to enable downstream analysis. Sequencing data were subsequently processed, and methylation information was extracted using the Tombo toolkit (https://nanoporetech.github.io/tombo). Methylation sites were detected via the DeepSignal-Plant [45] software and the results were annotated and graphically visualized. DMRs among different samples were determined using the Methylpy tool, with applied parameters including a maximum intersite distance of 500 bp (−dmr-max-dist 500), a minimum read coverage of 3 (−min-cov 3), and a requirement of no fewer than three differentially methylated sites to define a DMR (−min-num-dms 3).

### Differential methylation analysis

Differentially methylated cytosines (DMCs) and DMRs were identified through the application of Fisher’s exact test with Benjamini–Hochberg false discovery rate (FDR) correction [52]. Cytosines exhibiting an absolute methylation difference ≥0.2 and an FDR < 0.05 were designated as DMCs. The *S. rebaudiana* genome was partitioned into 1-kb bins to facilitate comparisons of methylation levels. Genomic regions meeting the criteria of FDR < 0.01 and absolute methylation differences of ≥0.2 (CG), ≥0.15 (CHG), or ≥0.1 (CHH) were categorized as DMRs.

### Transcriptome sequencing and analysis of DEGs

Total RNA was extracted from SrL, SrF, and SrS tissues collected at the initial flowering stage. Three biological replicates were prepared using the RNA prep Pure Plant Kit (DP441, Tiangen, China). RNA integrity and quality were evaluated using an IMPLEN NanoPhotometer spectrophotometer to ensure compliance with the required standards for downstream analyses. cDNA libraries were subsequently constructed from the qualified RNA samples, and sequencing was performed on the Illumina NovaSeq 6000 platform by Metware Biotechnology Co., Ltd. (Wuhan, China). Clean reads were mapped to the *S. rebaudiana* genome employing Hisat2 v2.0.4, and genes with an adjusted *P*-value <0.05 were designated as DEGs. A comparative transcriptome analysis across tissue samples was carried out, and KEGG pathway enrichment analysis was performed to assess the biological functions and pathways associated with DEGs.

### Bisulfite sequencing PCR validation

Bisulfite sequencing PCR (BSP) was performed to validate the methylation results from Nanopore sequencing, with genomic DNA extracted from the tissue using the CTAB method. DNA methylation treatment was executed using the DNA Methylation Kit provided by Cwbio Ltd. (Suzhou, China), while BS-PCR was conducted with DNA Polymerase from Vazyme Biotech Co., Ltd. (Nanjing, China). BSP primers are provided in Table S9. Amplified fragments were cloned into the pGreenII 62-SK vector for sequencing, and the resulting sequences were analyzed using CyMATE [53] software (http://www.cymate.org/).

## Supplementary Material

Web_Material_uhaf226

## Data Availability

The raw data, genome assemblies, and annotations have been deposited in the National Genomics Data Center (https://ngdc.cncb.ac.cn/) under accession number PRJCA028196. The RNA-seq data are accessible via the NGDC database at https://ngdc.cncb.ac.cn/ under accession number PRJCA016649.
