# Supplementary material for: Integrated genomic and DNA methylome analyses reveal epigenetic regulation of stevia glycoside biosynthesis in Stevia rebaudiana
Source: Hortic Res. 2025 Sep 2;12(12):uhaf226. doi: 10.1093/hr/uhaf226 (PMC12680500; doi:10.1093/hr/uhaf226)
Supplement: Web_Material_uhaf226 [file web_material_uhaf226.zip › Figure S1. Genome assembly and quality assessment of the S. rebaudiana..pdf]

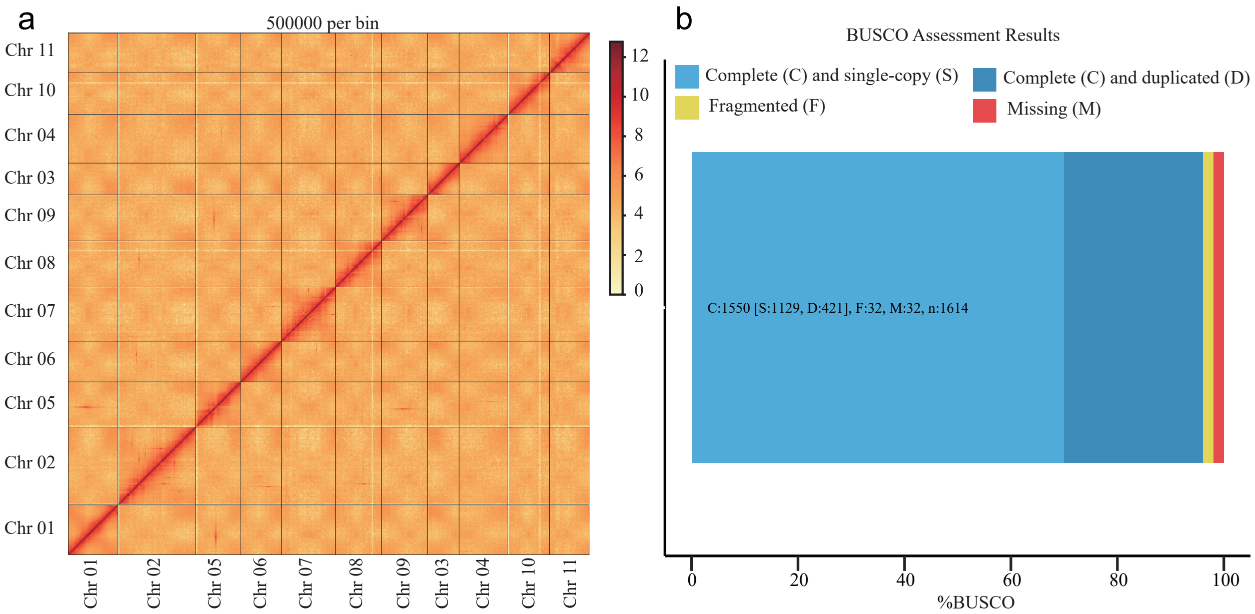

Figure S1. Genome assembly and quality assessment of the *S. rebaudiana*. (**a**) Heatmap of genomic interactions within *S. rebaudiana* chromosomes. (**b**) BUSCO assessment of the *S. rebaudiana* genome.
