# Supplementary material for: Integrated genomic and DNA methylome analyses reveal epigenetic regulation of stevia glycoside biosynthesis in Stevia rebaudiana
Source: Hortic Res. 2025 Sep 2;12(12):uhaf226. doi: 10.1093/hr/uhaf226 (PMC12680500; doi:10.1093/hr/uhaf226)
Supplement: Web_Material_uhaf226 [file web_material_uhaf226.zip › Figure S2. Gene family clustering analysis of five species..pdf]

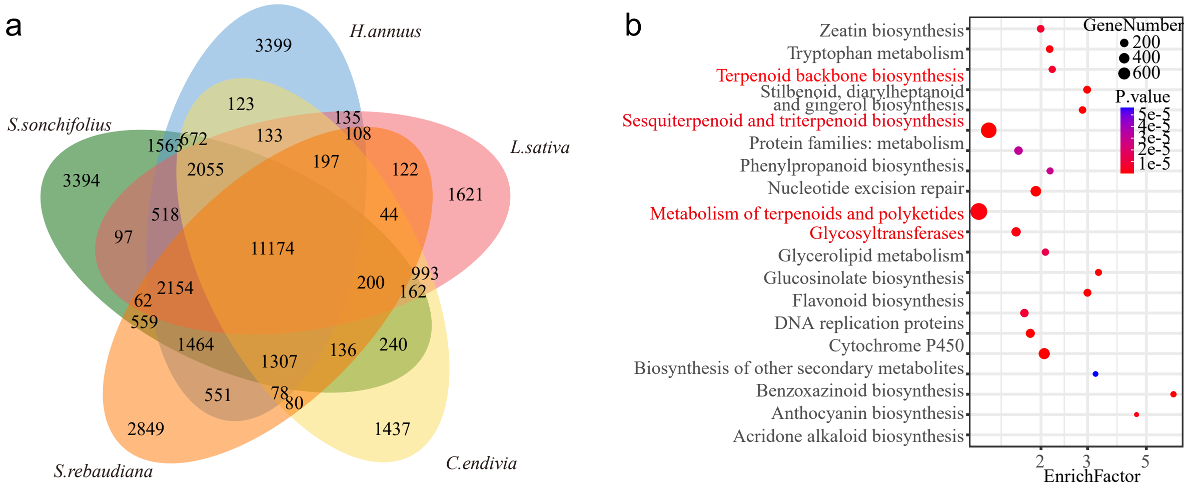

Figure S2. Gene family clustering analysis of five species. (**a**) Gene families across the five species are shown in a Venn diagram. (**b**) KEGG enrichment analysis of *S. rebaudiana*-specific genes families is presented.
