# Supplementary material for: Integrated genomic and DNA methylome analyses reveal epigenetic regulation of stevia glycoside biosynthesis in Stevia rebaudiana
Source: Hortic Res. 2025 Sep 2;12(12):uhaf226. doi: 10.1093/hr/uhaf226 (PMC12680500; doi:10.1093/hr/uhaf226)
Supplement: Web_Material_uhaf226 [file web_material_uhaf226.zip › Figure S3. Collinearity analysis among four species including S. rebaudiana..pdf]

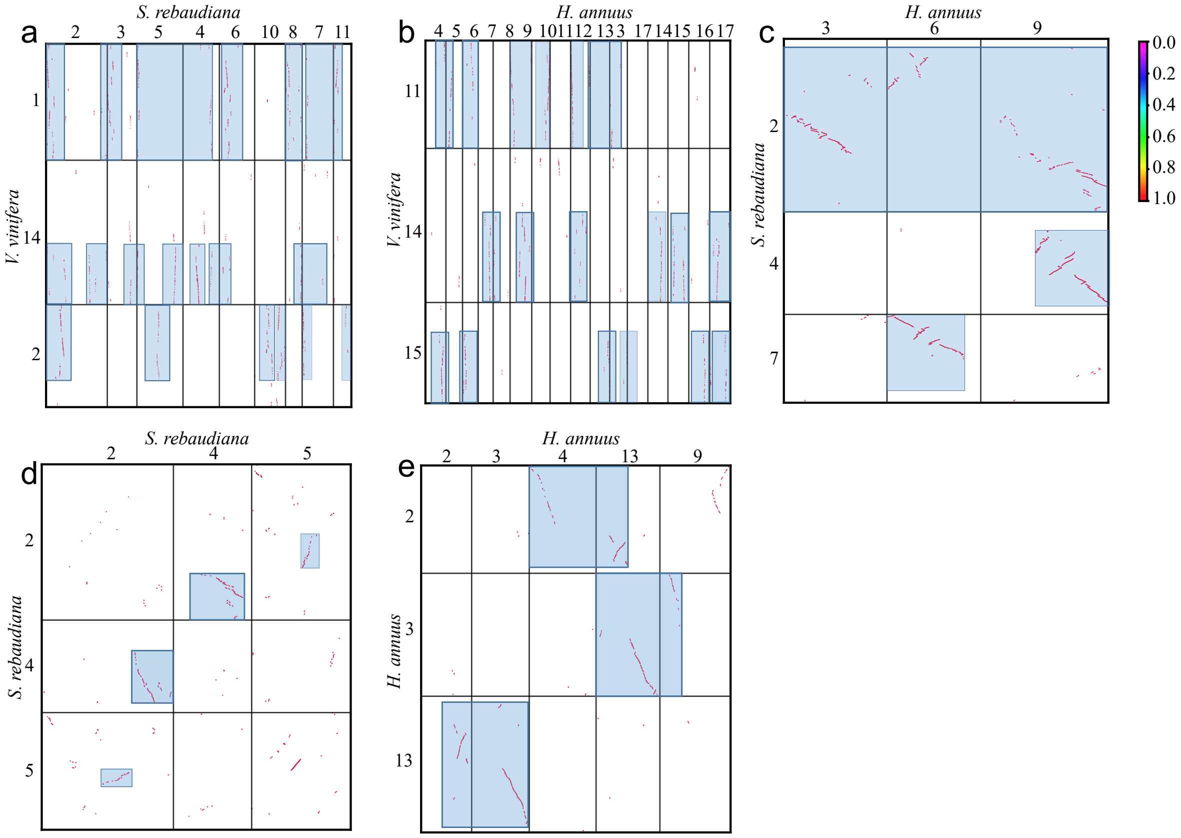

Figure S3. Collinearity analysis of *S. rebaudiana*, grape, and sunflower. (**a**) Intergenomic synteny block dot plots between the genomes of *V. vinifera* and *H. annuus*. (**b**) Intergenomic synteny block dot plots among the genomes of *V. vinifera* and *S. rebaudiana*. (**c**) Intergenomic synteny block dot plots among the genomes of *S. rebaudiana* and *H. annuus*. (**d**) Intra-genomic synteny block dot plots within the *S. rebaudiana* genome. (**e**) Intra-genomic synteny block dot plots within the *H. annuus* genome.
