# Supplementary material for: Integrated genomic and DNA methylome analyses reveal epigenetic regulation of stevia glycoside biosynthesis in Stevia rebaudiana
Source: Hortic Res. 2025 Sep 2;12(12):uhaf226. doi: 10.1093/hr/uhaf226 (PMC12680500; doi:10.1093/hr/uhaf226)
Supplement: Web_Material_uhaf226 [file web_material_uhaf226.zip › Figure S4. 5â_T-Aza inhibits the methyltransferases expression and promotes the SGs synthesis.pdf]

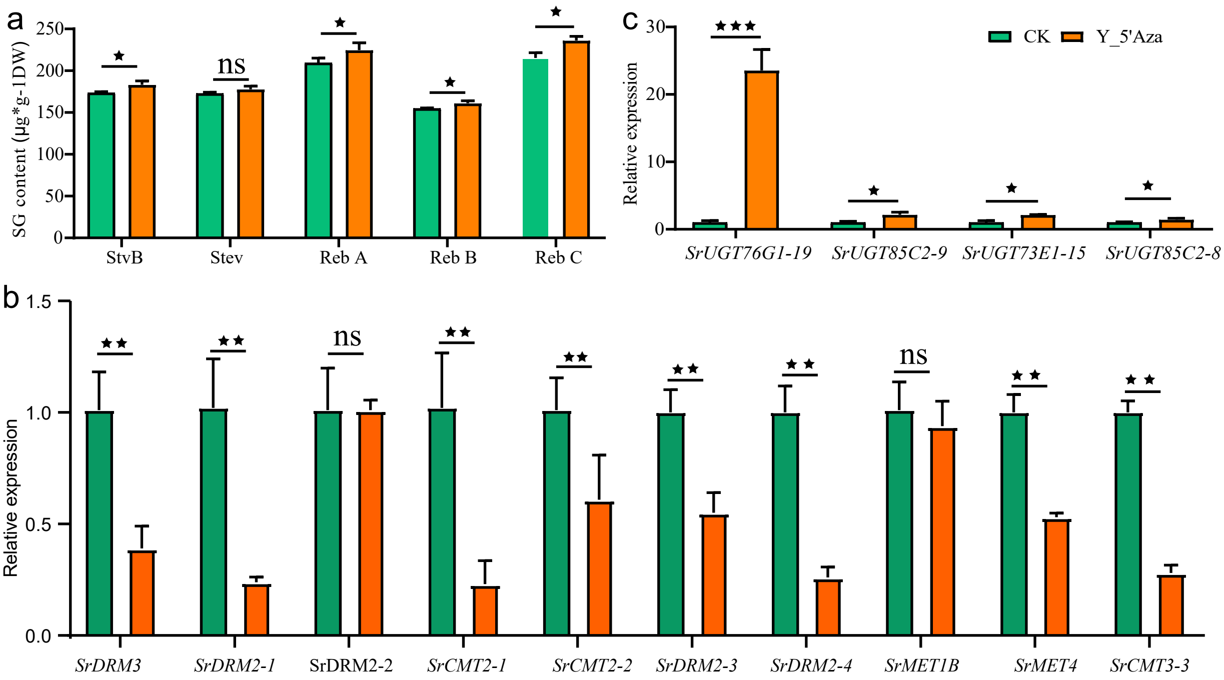

Figure S4. 5'-Aza inhibits the methyltransferases expression and promotes the SG synthesis. (**a**) The SG content of Stev B, Stev, Reb A, Reb B, and Reb C is quantified. (**b, c**) Relative expression levels of methyltransferases and glycosyl transferase are presented. Error bars represent mean ± SD (n = 3 biologically independent samples). Data in **b-c** subjected to Student's t-test with $p< 0.05$; DW: dry weight.
